# Cortico-Striatal-Thalamic Loop Circuits of the Salience Network: A Central Pathway in Psychiatric Disease and Treatment

**DOI:** 10.3389/fnsys.2016.00104

**Published:** 2016-12-27

**Authors:** Sarah K. Peters, Katharine Dunlop, Jonathan Downar

**Affiliations:** ^1^Institute of Medical Science, University of TorontoToronto, ON, Canada; ^2^Krembil Research Institute, University Health NetworkToronto, ON, Canada; ^3^Department of Psychiatry, University of TorontoToronto, ON, Canada; ^4^MRI-Guided rTMS Clinic, University Health NetworkToronto, ON, Canada

**Keywords:** corticostriatal, salience network, brain stimulation, depression, substance use disorders, anxiety disorders, repetitive transcranial magnetic stimulation

## Abstract

The salience network (SN) plays a central role in cognitive control by integrating sensory input to guide attention, attend to motivationally salient stimuli and recruit appropriate functional brain-behavior networks to modulate behavior. Mounting evidence suggests that disturbances in SN function underlie abnormalities in cognitive control and may be a common etiology underlying many psychiatric disorders. Such functional and anatomical abnormalities have been recently apparent in studies and meta-analyses of psychiatric illness using functional magnetic resonance imaging (fMRI) and voxel-based morphometry (VBM). Of particular importance, abnormal structure and function in major cortical nodes of the SN, the dorsal anterior cingulate cortex (dACC) and anterior insula (AI), have been observed as a common neurobiological substrate across a broad spectrum of psychiatric disorders. In addition to cortical nodes of the SN, the network’s associated subcortical structures, including the dorsal striatum, mediodorsal thalamus and dopaminergic brainstem nuclei, comprise a discrete regulatory loop circuit. The SN’s cortico-striato-thalamo-cortical loop increasingly appears to be central to mechanisms of cognitive control, as well as to a broad spectrum of psychiatric illnesses and their available treatments. Functional imbalances within the SN loop appear to impair cognitive control, and specifically may impair self-regulation of cognition, behavior and emotion, thereby leading to symptoms of psychiatric illness. Furthermore, treating such psychiatric illnesses using invasive or non-invasive brain stimulation techniques appears to modulate SN cortical-subcortical loop integrity, and these effects may be central to the therapeutic mechanisms of brain stimulation treatments in many psychiatric illnesses. Here, we review clinical and experimental evidence for abnormalities in SN cortico-striatal-thalamic loop circuits in major depression, substance use disorders (SUD), anxiety disorders, schizophrenia and eating disorders (ED). We also review emergent therapeutic evidence that novel invasive and non-invasive brain stimulation treatments may exert therapeutic effects by normalizing abnormalities in the SN loop, thereby restoring the capacity for cognitive control. Finally, we consider a series of promising directions for future investigations on the role of SN cortico-striatal-thalamic loop circuits in the pathophysiology and treatment of psychiatric disorders.

## Introduction

Psychiatric illnesses are among the leading causes of disability and disease burden worldwide in the 21st century. For example, the 2010 Global Burden of Disease study identified major depressive disorder (MDD) as the second leading cause of years of life lost to disability (Ferrari et al., 2013). Overall, mental and substance use disorders (SUDs) accounted for 22.9% of the global burden of years of life lost to disability (Whiteford et al., 2013), with a prevalence of around one billion cases worldwide (Whiteford et al., 2015). These illnesses have high chronicity and community burden, and often show low response rates to existing treatments. For example, in major depression, conventional interventions are ineffective in at least one-third of patients, and relapse rates are high even when remission is achieved (Rush et al., 2006; Ferrari et al., 2013). Thus, an important task for basic and translational neuroscience is to better understand the underlying pathophysiology of psychiatric illness, and to develop treatments that effectively target this pathophysiology.

Over the last 25 years, one of the major advances in the field has been the development of increasingly detailed maps of the neural pathways that are affected in psychiatric illnesses. Steady progress is being made in localizing abnormalities of both brain structure and brain function. The neurologist’s traditional question, “Where is the lesion?”, formerly had few well-defined answers for most psychiatric disorders. However, today there is at least a first approximation of an answer to this question for many of the most prevalent types of mental illness. Progress in localizing psychiatric neuropathology has come from advances in non-invasive neuroimaging techniques suitable for *in vivo* use in humans. These include structural imaging techniques for mapping gray and white matter pathology, such as voxel-based morphometry (VBM) and diffusion tensor imaging (DTI), as well as functional imaging techniques including functional magnetic resonance imaging (fMRI), and positron emission tomography (PET).

In healthy control subjects, application of these brain-imaging techniques has been especially fruitful for delineating the overall functional architecture of the human brain. One major discovery has been that brain activity, during tasks or at rest, is organized into functional networks of regions showing correlated activity over time. The networks themselves appear to be fairly consistent across individuals, with one influential report identifying a set of seven reliably reproducible major networks, subdivisible into a finer set of 17 smaller subnetworks (Yeo et al., 2011). The earliest description of resting-state networks was in the motor cortex (Biswal et al., 1995). Since that time, an extensive literature of thousands of publications has developed to describe the properties of several other major networks: a default-mode network most active during non-task cognitive states such as rumination or prospection (Raichle, 2015); a central executive network most active during performance of cognitive tasks involving attention or working memory (Bressler and Menon, 2010); and more circumscribed networks restricted to somatomotor or visual brain regions (Yeo et al., 2011).

**Figure 1:**
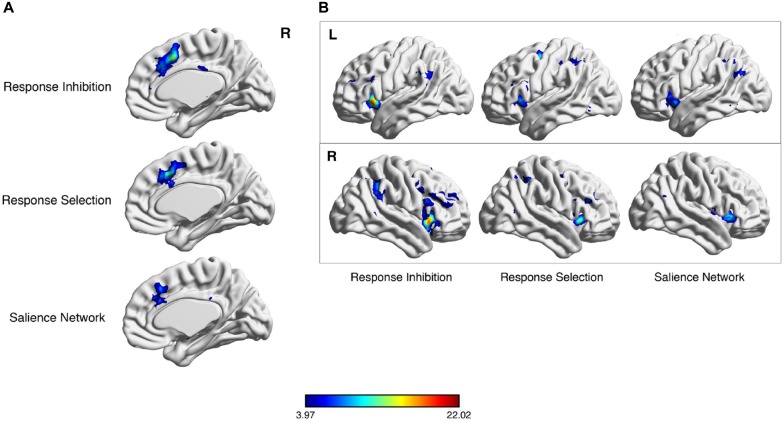
**Meta-analytic co-activation of salience network (SN) nodes for response selection and inhibition.** Neurosynth meta-analytic results following key word searches for “response inhibition”, “response selection” and “SN”. Considerable overlap exists in the dorsal anterior cingulate cortex (dACC) region **(A)** and lateral parietal cortex **(B)**.

Among these brain networks, one in particular is emerging as having particular significance to psychiatric illness: the salience network (SN; Seeley et al., 2007; Menon, 2011). Sometimes known by other terms such as the cingulo-opercular network (Dosenbach et al., 2008), the SN corresponds to the more anterior of the two subnetworks of the “ventral attention network (VAN)” described by Yeo et al. (2011). The SN features core nodes in the dorsal anterior cingulate cortex (dACC) and the bilateral anterior insula (AI), as well as additional cortical notes in specific regions of the dorsolateral prefrontal cortex (dlPFC) and inferior parietal lobule (IPL; Figure 1). In addition to these cortical nodes, the SN also includes a specific set of subcortical nodes in the head of the caudate nucleus, the mediodorsal nucleus of the thalamus (MDN) and dopaminergic brainstem nuclei (Menon, 2011). Together, these structures complete a discrete cortico-striatal-thalamic-cortical (CSTC) loop that can be discerned using structural or functional neuroimaging (Seeley et al., 2007; Metzger et al., 2010), and that also corresponds to an analogous loop evident on tract-tracing studies in nonhuman primates (Choi et al., 2016).

**Figure 2:**
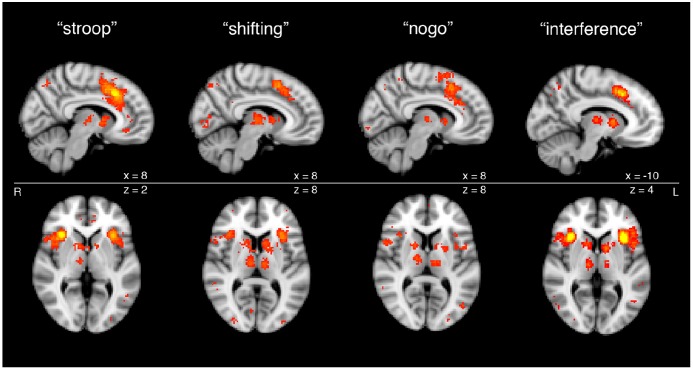
**Cortical and subcortical nodes of the SN from meta-analyses of functional magnetic resonance imaging (fMRI) studies of task-based activation during cognitive control.** Subcortical components of SN processing are apparent across a variety of executive functioning tasks, including Stroop task, set shifting task, Go-No Go task and Flanker interference task, as shown through meta-analytic functional imaging data.

In healthy brain function, cortical nodes of the SN may be activated passively by “salient” sensory stimuli (i.e., stimuli that draw attention for being unexpected, novel or behaviorally relevant; Corbetta et al., 2000; Downar et al., 2000, 2001, 2002). These cortical activations become accompanied by subcortical activations of the entire cortico-striatal-thalamic loop circuit during active, voluntary engagement of cognitive control, response selection or response inhibition; common examples include the Stroop task, Go/No-Go task or flanker interference task (Figure 2). As such, the SN has been proposed to play a key role in cognitive control (Ham et al., 2013), i.e., switching brain activity between introspective, ruminative functions of the default-mode network and externally focused, task-based functions of the central executive network (Menon and Uddin, 2010).

The importance of the SN to psychiatric illness emerges from an influential meta-analysis of 193 VBM studies enrolling more than 7000 individuals with a wide variety of psychiatric diagnoses including depression, bipolar disorder, schizophrenia, SUD, obsessive-compulsive disorder (OCD) and anxiety disorders (Goodkind et al., 2015). Examining the patterns of decreased gray matter in each disorder, the authors found a common substrate across all diagnoses: loss of gray matter in the dACC and bilateral AI. The loci of gray matter loss corresponded closely with the core cortical nodes of the SN. As such, loss of structural and functional integrity in the SN, and a resultant impairment of cognitive control, has been proposed as a transdiagnostic feature across many psychiatric illnesses (McTeague et al., 2016) and also as a target for novel therapeutic interventions, such as brain stimulation (Downar et al., 2016).

As of this writing, over 1500 publications have made reference to the SN, and a number of excellent reviews are available to summarize this literature (Menon and Uddin, 2010; Menon, 2011; Dutta et al., 2014), including a recent book devoted entirely to the subject (Uddin, 2017). The present review article aims to build upon this work by considering the SN not just as a resting-state cortical network, but more specifically as an integrated, CSTC network with a particular role in the voluntary engagement of cognitive control. First, we examine the anatomy and function of the SN-CSTC in the healthy state. Second, we review the available literature on abnormalities of the SN-CSTC across a range of psychiatric disorders. Third, we review the available literature on how brain stimulation techniques can modulate the activity of the SN-CSTC, and how this modulation may achieve therapeutic effects in psychiatric illness. Finally, we consider a series of promising directions for future investigations on the role of the SN-CSTC loop circuits in the pathophysiology and treatment of psychiatric disorders.

## Anatomy and Function of the SN Cortico-Striatal Loop

### Anatomy of the SN Cortico-Striatal-Thalamic Loop

The cortical nodes of the SN are evident in one of the 17 resting-state networks cataloged in the 1000-subject, connectivity-based parcellation of resting-state fMRI data by Yeo et al. (2011). This network is the more anterior of the two subnetworks that comprise the larger VAN. While the more posterior subnetwork includes posterior insula and mid-cingulate nodes, the more anterior subnetwork includes adjacent AI and dACC regions (Figure 1). In addition, this network includes nodes in a specific region of the dlPFC, on the middle frontal gyrus, distinct from other lateral prefrontal regions assigned to the “frontoparietal” and “dorsal attention” networks in the Yeo et al. (2011) catalog. The SN also includes a specific region of the IPL, in the angular gyrus.

The interested reader can readily perform an independent replication of this map of regions by using the online, automated meta-analytic tool Neurosynth (Yarkoni et al., 2011; Figure 2). Entering the term “SN” yields a set of cortical regions that overlaps very closely with the more anterior cingulo-insular network of Yeo et al. (2011). Entering the term “cognitive control”—a core domain within the National Institute of Mental Health’s proposed Research Domain Criteria for psychopathology research (Insel, 2014)—or associated terms for the subdomains of “response inhibition” or “response selection” likewise yields very a similar set of regions. Note that the purpose of this exercise is not to infer a particular functional role for these regions, as such inferences, based on correlations alone, are vulnerable to validity challenges. Instead, this meta-analytic exercise demonstrates the consistent co-activation of these regions as a coherent network across a variety of studies in the neuroimaging literature, with the functions of this network to be discussed subsequently.

The meta-analytic maps in Figure 2 are also notable because they reveal distinct subcortical nodes co-activating with the cortical nodes of the SN during the performance of active tasks, as opposed to during the resting state, from which the maps of Yeo et al. (2011) were derived. These subcortical nodes are present in the head of the caudate nucleus bilaterally, as well as in the thalamic MDN. These striatal and thalamic nodes, taken together with the cortical nodes of the SN, comprise the complete and functionally integrated CSTC loop circuit particular to the SN, for the purposes of the remainder of our review article.

### The SN CSTC Loop Circuit in its Classical Neuroanatomical Context

The classical description of cortico-striatal-thalamo-cortical loop circuits of Alexander et al. (1986) included five parallel, functionally segregated circuits: (i) a motor CSTC loop through the supplementary motor area, putamen, ventrolateral globus pallidus interna (GPi) and ventrolateral thalamus; (ii) an oculomotor CSTC loop from the frontal eye field to the body of the caudate, caudomedial GPi and lateral ventral anterior thalamus; (iii) a lateral orbitofrontal CSTC loop from the lateral orbitofrontal cortex to the ventromedial caudate, medial dorsomedial GPi and medial ventral anterior thalamus; (iv) a dorsolateral prefrontal CSTC loop from the dlPFC to the dorsolateral caudate, lateral dorsomedial GPi and parvocellular mediodorsal thalamus; and (v) an anterior cingulate CSTC loop from the dorsal ACC to the ventral striatum, ventral pallidum and posteriomedial mediodorsal thalamus.

This classical set of five CSTC loops was originally derived from nonhuman primate work; in humans, a more complex and numerous set of CSTC loops is now considered likely. Indeed, a 1000-subject parcellation of resting-state functional connectivity in the striatum rather than the cortex (Choi et al., 2012) revealed substantially more than five distinct striatal regions, each associated with a specific cortical network cataloged in the parcellation of Yeo et al. (2011). As such, the CSTC loop of the SN may be expected to contain features of multiple classical loop circuits, rather than corresponding precisely to just one of them. The SN loop contains nodes that appear in the classical dorsolateral prefrontal CSTC loop (i.e., dlPFC, dorsolateral caudate and mediodorsal thalamus nodes), but also contains cortical nodes in the anterior cingulate and the AI, which do not appear in any of the classically described loops.

Conversely, each of the classically described CSTC loops of Alexander et al. (1986) also includes a specific subregion of the substantia nigra: rostrolateral for the dlPFC, and rostrodorsal for the anterior cingulate. Substantia nigra activations do not appear in the meta-analytic renderings of the SN (Figure 2), as may be expected given the limited spatial resolution of the fMRI data from which these renderings are derived. However, it should be noted that the substantia nigra and other midbrain dopaminergic centers such as the ventral tegmental area (VTA) are important contributors to all CSTC loops, and that rostral substantia nigra cell populations have been identified as key contributors to the CSTC loops serving SN cortical regions even in classical descriptions (Alexander et al., 1986).

### Functional Roles of Specific SN-CSTC Nodes

Understanding the importance of SN function in cognitive control requires a thorough review of the broader functional roles of the SN’s individual cortical and subcortical components. As noted earlier, key pre-Rolandic cortical nodes of the SN include the bilateral AI, dACC and dlPFC (Seeley et al., 2007). Separately, each of these areas have been linked to self-awareness, body perception (Craig, 2009) and fundamental cognition (Delevich et al., 2015). The AI plays an integral role in the response and experience of emotional states (Craig, 2003), and level of activity has been shown to correlate with stimulus valence (Anders et al., 2004). In addition to emotional perception, the AI is proposed to estimate changing environmental demands to modulate flexible cognitive control (Jiang et al., 2015). The AI also responds during action selection during decision-making (Paulus and Stein, 2006), and is therefore an integral component of cognitive control and the SN. Furthermore, the dACC has separately been implicated in cognitive regulation (Delevich et al., 2015), divergent thinking, error detection and response selection (Abraham et al., 2012; Sun et al., 2016). The dlPFC has extensive projections to striatal nuclei and is involved in top-down modulation of goal-directed behavior (Furman et al., 2011). The dlPFC is also a key hub of cognitive control, as it is implicated in executive function (Kuo and Nitsche, 2012) and has been identified as a dominant cortical area within the brain’s central cognitive control network (Menon, 2011; McTeague et al., 2016).

Co-activation among cortical regions of the SN is also associated with cognitive and behavioral phenomena related to decision-making and cognitive control. For one, the AI, dACC and dlPFC activate synchronously in response to uncertainty in the environment; these regions overlap with areas implicated in negative mood states (Feinstein et al., 2006; Naqvi and Bechara, 2009; Davis and Hasson, 2016). The dACC and AI activate together during decision-making; co-activation has been shown to increase with task difficulty and stimulus ambiguity. This finding suggests that the “difficulty dependent functional architecture” between the dACC and AI plays a role in cognition by filtering and integrating internal and external stimuli during cognitive tasks (Lamichhane et al., 2016). In addition to coactivity with separate functional networks, the dACC also possesses extensive cortico-cortical connections within the PFC, including the cognitive dlPFC hub and premotor regions, placing it at the crossroads of learning and behavior systems (Haber, 2016).

Like other networks containing regions of association cortex, the SN links to subcortical nodes in areas of the striatum and limbic system, including (as noted above) the MDN, the dorsal striatum and dopaminergic nuclei within the midbrain and brainstem. The striatum, along with the entire basal ganglia, is important in coordinating and sequencing the diverse functions of the frontal lobes, from goal formation to executive function to cognition and the selection of specific actions and movements (Schultz et al., 2003). Whereas the ventral striatum is involved in reward and motivation, the central and dorsal striatum—including the caudate nucleus and the putamen—play more integral roles in cognition and executive function (Haber, 2016).

Within the thalamus, the MDN is a relay nucleus with reciprocal connections to regions of the medial PFC (mPFC) that assists in flexible action selection by integrating information from cortical, limbic and basal ganglia regions (Delevich et al., 2015). Loss of functional communication between the MDN and mPFC due to physical or chemical lesions has been shown to interrupt behavioral flexibility (Parnaudeau et al., 2013), learning and decision-making in both humans and animals (Mitchell, 2015). The MDN is integral to rapid associative learning and other executive tasks that involve complex cognition, though its precise role in integration and cognition remains incompletely characterized (Mitchell, 2015).

The role of the SN as a coordinator of other networks for cognitive control is supported by coactivation of its cortical and subcortical nodes during tasks. For example, functional links between the insula, which predicts changing cognitive control demand, and “classic” cognitive control circuits rooted in the dACC and dlPFC, allow for reactive attentional control (Jiang et al., 2015). Extensive connections from these regions to the dorsal striatum may link reactional cognitive control to behavioral guidance, allowing for on-line behavior regulation in response to salient environmental stimuli (Botvinick et al., 2004; Haber, 2016). Specifically, the dACC projects primarily to the dorsal caudate nucleus and ventral striatum, overlapping partially but not completely with frontostriatal projections from the dlPFC (Haber, 2016). Strengthened resting state functional connectivity between the AI and dACC has been associated with enhanced cue reactivity in other brain areas including the putamen, suggesting that functional connections throughout the loop allow incoming information to exert downstream effects on modulatory striatal areas (Janes et al., 2015). Although a general topographic organization exists between the frontal cortex and subcortical targets, there exists a complex convergence across CSTC loops that originate in prefrontal areas, including the ventromedial PFC (vmPFC) and orbitofrontal cortex; this overlap suggests that CSTC circuit integration is structural as well as functional, and provides modulation between and across reward, prediction and saliency circuits (Averbeck et al., 2014; Haber, 2016).

At the level of the brainstem, dopaminergic regions also play a significant modulatory role in the SN CSTC loop. Dopamine is integral to SN function: as noted earlier, the VTA and rostral substantia nigra project to the basal ganglia regions that subserve the classically described CSTC loops serving the anterior cingulate and dlFPC; these dopaminergic projections consequently play important roles in SN activity and modulation. Dopamine projections throughout the SN have been noted to play important roles not only in reward-oriented learning and goal-directed behavior, but also in processing motivational stimuli by directing attention to positive, adaptive or rewarding environmental stimuli (i.e., mediating the salience of environmental stimuli; Berridge, 1996; Koob and Volkow, 2010; Kroemer et al., 2014). Dopaminergic neurons in the midbrain are critical to CSTC loops that encompass integrated learning, executive function and motor control. Importantly, the mesolimbic and nigrostriatal dopaminergic pathway have been implicated in the encoding of “saliency prediction errors”, underscoring their broader contributions to the attribution of salience to environmental stimuli (Kapur, 2003; Haber, 2016).

### An SN Architecture for Salience, Cognitive Control and Response Selection

In summary, the SN CSTC loop appears to function as a distinct, well-integrated regulatory circuit that links cortical and subcortical regions involved in cognition, attentional control, motivation, motor control and salience. The SN CSTC loop has been described as a cortical input “filter” that selectively identifies and flags stimuli on which to base cognitive and behavioral responses (Furman et al., 2011; Choi et al., 2012). The dlPFC primarily projects to the dorsolateral caudate to serve cognitive control and executive functioning (Furman et al., 2011), while the thalamic MDN supports cortico-striatal-cortical information transfer and modulates cortical activity through extensive connections with the PFC and midbrain dopaminergic regions (Mitchell, 2015). Dopaminergic nuclei in the midbrain and brainstem, which are often associated with limbic circuitry, project widely throughout multiple loops of the striatum to serve a variety of functions, both structurally and functionally. For example, the substantia nigra makes extensive connections with the striatum, cortex, thalamus and neighboring brainstem regions, allowing dopaminergic outputs to exert far-reaching effects on the flow of CSTC information (Haber, 2016). At the same time, however, distinct populations of neurons in the rostral substantia nigra may project more specifically to the CSTC loops serving the cortical regions of the SN, as recognized in classical descriptions of CSTC loop architecture (Alexander et al., 1986). Working together, the subcortical components of the SN communicate extensively with their cortical counterparts to select salient (motivationally relevant) stimuli, and to enable such stimuli to direct cognition and behavior. It is this architecture that enables the SN to play its central roles in salience detection, cognitive control and the selection and/or inhibition of behavioral responses during healthy brain function.

## Abnormalities of SN-CSTC Loop Circuits in Psychiatric Illnesses

Aberrations of corticostriatal loop circuits that modulate cognitive control and goal-directed behavior may underlie the pathophysiology of several psychiatric illnesses. Although specific psychiatric disorders, such as depression and schizophrenia are characterized by distinct constellations of symptoms, structural and functional abnormalities appear throughout similar nodes and networks transdiagnostically, across many Axis I disorders (Goodkind et al., 2015). It is possible that a spectrum of SN aberrations, ranging from hypo- to hyperactivity through the CSTC loop, can trigger a broad range of disabling behavioral and cognitive symptoms that are reflective of disrupted cognitive-attentional processing. The following section briefly explores the potential involvement of such corticostriatal circuits in the etiology, manifestation and treatment of major psychiatric disorders, including MDD, SUD, anxiety disorders including OCD and post-traumatic stress disorder (PTSD), schizophrenia and eating disorders (ED).

### Major Depressive Disorder (MDD)

MDD is characterized by persistent low mood, accompanied by loss of interest and pleasure, increased fatigue and irritability and difficulty concentrating and making decisions (Benazzi, 2006). Neuroimaging analyses of individuals with MDD have revealed both anatomical and functional differences throughout SN nodes relative to healthy controls. Anatomically, investigations have revealed reduced gray matter volume in the anterior cingulate cortex, caudate nucleus, putamen (Bora et al., 2012; Shepherd, 2013). Thalamic volume abnormalities are mixed (Webb et al., 2014; Zhao et al., 2014; Hagan et al., 2015). Neurochemical ligands via PET have shown reduced midbrain and subcortical serotonergic receptor binding in MDD (Hahn et al., 2014; Yeh et al., 2015).

While anatomical and neurochemical differences have been identified in SN nodes, symptoms of MDD have often been attributed to dysfunctional ventral CSTC loops rather than volumetric deviations alone (Bora et al., 2012; Kerestes et al., 2015). MDD patients generally exhibit reduced sensitivity to reward; this cognitive blunting is reflected by reduced ventral CSTC loop activation in the orbitofrontal cortex, anterior cingulate and ventral striatum to reward and loss receipt/anticipation (Smoski et al., 2011; Chantiluke et al., 2012; Schiller et al., 2013; Admon et al., 2015; Manelis et al., 2016).

Although MDD has classically been described in terms of dysfunctional ventral corticostriatal loops that play a role in affect and reward, recent reports have identified abnormalities within dorsal, cognitive corticostriatal loops in MDD patients (Kerestes et al., 2015). Specifically, individuals with depression are reported to show increased functional connectivity between the dorsal caudate nucleus and right dlPFC, with disease severity correlated to increased connectivity; hyperactivation between these regions may indicate a pathological compensation of cognitive processing over negative emotions and stimuli (Furman et al., 2011; Kerestes et al., 2015).

In addition to SN structural and functional aberrations in MDD, symptomatic improvement to conventional treatment is predicted by differential SN function. Connectivity between the putamen, caudate and cingulate during a monetary incentive delay task has been shown to predict response to psychotherapy (Admon et al., 2015; Walsh et al., 2016). High baseline ACC and low striatal activity were also shown to be predictive of improvement on antidepressants in a recent meta-analysis of 15 studies (Fu et al., 2013).

SN function change is also attributed to symptomatic improvement to conventional interventions. A recent meta-analysis based on nine MDD fMRI studies established that antidepressant treatment resulted in increased activation in the dlPFC, AI and ACC, and decreased activation in the thalamus and caudate nucleus, during emotional processing, representing normalization of resting state activity (Delaveau et al., 2011). Similar analyses consistently identify functional increases in these areas relative to baseline following SSRI treatment based on functional activity during negative mood induction (Fitzgerald et al., 2008) and incentive cue processing (Stoy et al., 2011).

MDD patients vary widely in terms of symptom presentation; recent work has sought to characterize MDD by distinct symptomatic and neural subtypes, or “endophenotypes”. In one notable study, MDD was partitioned into three subtypes, one of which was characterized by regulatory deficits in cognitive control and altered electrical current densities in nodes of the SN, specifically the dlPFC and dACC (Webb et al., 2016). The conflation of several hypothetically heterogenous MDD pathophysiological profiles in functional studies to date may obscure important characteristics that differentiate one or more of these subtypes. Future work should further characterize the symptomatic and neural heterogeneity of MDD into reliable endophenotypes.

### Substance-Use Disorders (SUDs)

SUDs involve abnormal reward and motivational responses to salient drug-related cues in the environment. Impulsivity and compulsivity are well-characterized traits of SUD, and both traits play important roles in the addiction cycle (Koob and Volkow, 2010). Traditional neurobiological research in SUD has attempted to identify how subcortical and dopaminergic systems affect drug seeking behavior (Menon, 2011). For example, combined PET-fMRI data suggests that frontostriatal abnormalities may be associated with a reduction of striatal D2 dopamine receptors and prefrontal, dlPFC metabolic activity (Tomasi and Volkow, 2013).

Structurally, nodes of the SN display volumetric abnormalities in SUD. For example, cocaine users display altered cingulate, insula and caudate volume (Ersche et al., 2011; Moreno-López et al., 2012). Also, methamphetamine craving was associated with volumetric differences in the insula, PFC and thalamus; this association was further correlated with dopaminergic receptor availability in the midbrain (Morales et al., 2015). Functional abnormalities of single SN nodes have been related to SUD behaviors; the AI is considered critical to conscious drug-seeking motivation (Naqvi and Bechara, 2009).

SN network connectivity is implicated in SUD. For example, lower resting-state functional connectivity between the striatum, cingulate and insula have been found in cocaine users; dorsal ACC and dlPFC connectivity was linked to loss of control and impulsivity, respectively (Hu et al., 2015). Furthermore, reduced activation in the dorsal caudate and dlPFC has been found to be correlated with impulsivity and Stroop task errors in SUD (Qiu et al., 2013; Feng et al., 2016; Yuan et al., 2016). Conversely, gambling addiction has displayed increased PFC connectivity to the striatum; this connectivity was associated with impulsivity, smoking frequency and craving severity (Koehler et al., 2013). Stronger functional coherence within SN cortical nodes was recently linked to enhanced reactivity to smoking cues in individuals with nicotine dependance, suggesting that hyperconnectivity within the SN may be responsible for increased cue reactivity (Janes et al., 2015).

On task-based fMRI, another study used a reward-guided decision-making task to investigate reward prediction errors—signals that guide learning behaviors—in men with alcohol addiction. SUD patients also demonstrated abnormal dlPFC-striatal functional connectivity following “wins”, and “losses”, suggesting that learning mechanisms are impaired due to disrupted regulation of frontostriatal interactions (Park et al., 2010). Similarly, reduced response to reward is observed across the striatum, mPFC and dlPFC (Forbes et al., 2014).

Additionally, it appears that addictive substances activate dopamine-dependent saliency systems in the brain, resulting in the replacement of adaptive stimuli by more salient drug-related cues (Koob and Volkow, 2010). For example, higher dorsal caudate reactivity during cue induced craving has been found in heavy drinkers, while ventral striatal activity was found in lighter drinkers, and this the functional difference between drinkers was correlated with compulsive craving severity (Vollstädt-Klein et al., 2010). In another study, higher caudate and ACC activity during cue-induced craving predicted a transition to heavier drinking habits (Dager et al., 2014). Increases in compulsive drug-seeking behavior has been hypothesized to represent a shift from “top-down”, prefrontal cortical behavior control to striatal behavioral control due to progressively altered dopamine transmission (Goldstein et al., 2009; Koob and Volkow, 2010).

In summary, drug addiction has been described as a process of maladaptive neuroplasticity that begins in mesolimbic reward circuits and emanates through interconnected loops in the dorsal striatum, eventually cascading to the cortex where dysregulation results in permanent changes to habitual behavior and impulsivity (Koob and Volkow, 2010). SN frontostriatal circuit integrity may be associated with improved impulse control (Peper et al., 2013); conversely, improved top-down cognitive control over the striatum by prefrontal cortical regions may reduce impulsivity (Peters and Büchel, 2011).

### Obsessive-Compulsive Disorder (OCD)

OCD is classically characterized by intrusive thoughts (obsessions) that are distressing and disruptive to normal function, followed by repetitive actions (compulsions) aimed at reducing the associated distress (American Psychiatric Association, 1994). In more than half of individuals with OCD, these symptoms may be severe enough to cause absence from work, school or social engagements; further, treatment of these severe cases is often more difficult (Bourne et al., 2012). Importantly, obsessions and compulsions represent dysfunctional cognitive and motor processes that become repetitive and disturbing; it is suggested that hyperactivation within CSTC circuits and changes to CSTC topography may underlie such ritualistic thoughts and behaviors (Graybiel and Rauch, 2000; Nakamae et al., 2014).

OCD has traditionally been attributed to dysfunction within orbitofronto-CSTC loops; despite being somewhat distinct from CSTC loops that traditionally mediate the cognitive domain, this dysfunction is hypothesized to contribute to the unusual cognitive control of behavioral patterns often observed in OCD (Graybiel and Rauch, 2000; Dunlop et al., 2016). CSTC loop circuits are also relevant to OCD due to their integral role in emotional, cognitive and behavioral self-regulation (Marsh et al., 2009a). Results of a 7-study meta-analysis examining OCD resting-state fMRI suggested OCD tends to most reliably demonstrate corticostriatal network abnormalities relative to other anxiety disorders (Peterson et al., 2014). While many symptoms of OCD are related to disruptions within orbitofronto-CSTC loops, regions of the SN likely play a large role in specific symptoms; OCD symptoms.

Within nodes of the SN, volumetric changes have been identified in the ACC, striatum and thalamus (Gilbert et al., 2008; Hoexter et al., 2012; Hou et al., 2013; Atmaca et al., 2016). Functionally, the ACC appears to be abnormally altered during disease-relevant tasks. For example, OCD patients experience ACC hyperactivity during symptom aggravation and while committing errors during a flanker interference task (Bourne et al., 2012). Even outside of active task engagement, nodes of the SN are abnormally hyperactive: increased resting state functional activity of the ACC, MDN (Rauch et al., 2006a; Bourne et al., 2012), dlPFC (Figee et al., 2014) and caudate nucleus (Graybiel and Rauch, 2000) has been noted in individuals with OCD. Frontostriatal functional connectivity stemming from the dlPFC (Vaghi et al., 2016), and altered striatal connectivity to the insula (Bernstein et al., 2016) has been linked to OCD severity.

Interestingly, functional changes within SN nodes are related to OCD symptom improvement. Notably, stereotaxically targeted lesions of the dACC during cingulotomy procedures results in symptom improvement in some individuals with treatment-resistant OCD (Dougherty et al., 2002); such lesions also reduce glucose metabolism in the caudate, medial dorsal thalamus and caudate (Zuo et al., 2013). Traditional pharmacotherapies are shown to attenuate ACC and striatal hyperactivity (Rauch et al., 2006a), and normalize thalamic, cingulate and putamen gray matter volume (Hoexter et al., 2012; Atmaca et al., 2016). On resting-state fMRI, reduced dorsal caudate-anterior thalamus functional connectivity was associated with symptomatic improvement following fluoxetine (Anticevic et al., 2014). Such findings highlight the importance of SN-CSTC dysfunction in the pathophysiology of OCD.

### Post-Traumatic Stress Disorder (PTSD)

PTSD occurs in approximately 10% of individuals who have experienced a traumatic event; symptoms include intrusive re-experiencing of the traumatic event, and avoidance behaviors in order to escape similar or triggering situations (Koch et al., 2016). Changes in arousal, behavior and mood, as well as impairments in executive functioning, are also commonly seen in individuals with PTSD (American Psychiatric Association, 2013). Structurally, PTSD patients exhibit reduced gray matter volume in SN regions including the ACC, striatum and insula (Meng et al., 2016); caudate and insular volumetric reductions correlate with PTSD severity (Herringa et al., 2012). Cortical thinning has also been observed in the rostral ACC and insula, and disrupted structurally connectivity is seen between the ACC, thalamus and insula in PTSD (Mueller et al., 2015).

There is evidence that symptoms associated with PTSD involve potential changes to both singular SN nodes and corticostriatal circuits. First, during symptom provocation, PTSD symptom severity correlates with ACC activity, and heightened activity variability in the insula and ACC, and lower variability in activity in the dlPFC and striatum (Ke et al., 2015). Another study also found increased connectivity between the insula, mPFC, striatum and dorsal ACC during symptom provocation (Cisler et al., 2014). Second, generalization of fear associations in PTSD is associated with heightened insula and thalamus activity (Morey et al., 2015). Third, PTSD severity during an emotional Stroop task was associated with higher dlPFC, dmPFC and dorsal ACC activation to emotional stimulation (White et al., 2014). Finally, higher dorsal caudate and frontal activation during inhibitory control predicts response to CBT in PTSD, suggesting a role of SN function in symptom improvement following psychotherapy (Falconer et al., 2013).

Resting state functional connectivity also has revealed abnormal SN functional connectivity in PTSD. One recent meta-analysis showed that PTSD patients display enhanced connectivity within the SN, suggesting excessive salience processing within the SN serves as a mechanism of abnormal hyper-monitoring the external environment (Koch et al., 2016). Similarly, individuals with PTSD demonstrate hyperactivity relative to controls within the AI and dACC across a variety of executive functioning and emotional processing tasks, in conjunction with reduced functional connectivity between regions involved in fear, stress and negative affect. It is possible that enhanced vigilance combined with decreased top-down cognitive control over fear responses underlies PTSD symptoms (Rauch et al., 2006b; Patel et al., 2012).

With regards to abnormalities throughout CSTC loops, individuals with PTSD have been shown to have reduced thalamic functional connectivity with a variety of brain regions including the rostral ACC and mPFC (Duarte et al., 2011; Yin et al., 2011), and it has been suggested that flashback symptoms occur in part due to dysfunctional corticothalamic activity (Liberzon et al., 1996/1997). Recently, the medial rostral dorsal caudate was identified as a site of convergence between the IPL, dlPFC and dACC, suggesting that striatal inputs that mediate and bias attentional salience and cognitive control (Choi et al., 2016). A region allowing for interaction of such cortical areas may be strongly implicated in the allocation of stimuli salience and environmental attention, and authors suggest that future investigations probe this area to learn more about its involvement and potential therapeutic value in disorders including PTSD (Choi et al., 2016).

### Schizophrenia

Schizophrenia is a disabling psychiatric condition that manifests in primarily three domains: positive, negative and cognitive symptoms. Positive symptoms include additions to an individual’s repertoire of thoughts or behavior, such as hallucinations or delusions, whereas negative symptoms refer to flattened affect, anhedonia or catatonic symptoms (American Psychiatric Association, 2013). Individuals with this disorder may experience impairments in executive function and have been found to have deficits in cognitive, but not motor, learning (Foerde et al., 2008; Parnaudeau et al., 2013). In addition to cognitive deficits, schizophrenia is defined by psychosis and psychotic episodes (American Psychiatric Association, 2013).

Structural abnormalities within the SN have been observed in schizophrenia, specifically between the insula, ACC, dlPFC and striatum (White et al., 2010; Menon, 2011; Quan et al., 2013; Iwabuchi et al., 2015; Chen et al., 2016). Cortical thinning is also observed in the AI, IFG and ACC; this volumetric change is reflected by altered functional connectivity (Pu et al., 2012; Pujol et al., 2013). Further, schizophrenia has been associated with abnormal dopamine signaling within the cortex (Knable and Weinberger, 1997).

Psychosis and salience attribution deficits in schizophrenia are related to inter-network dysfunction between the SN, CEN and DMN (Palaniyappan et al., 2011, 2013; Moran et al., 2013; Lee et al., 2014; for a comprehensive review, see Nekovarova et al., 2014). Patients experiencing auditory hallucinations show increases in AI and frontal operculum activation and altered SN dynamics, indicating that internally generated stimuli are perceived as abnormally salient (Sommer et al., 2008; Lefebvre et al., 2016). Indeed, AI resting-state functional connectivity is correlated to symptom severity (Manoliu et al., 2013).

Frontostriatal dysconnectivity between the dlPFC, dACC, AI and putamen are observed during an emotion judgment task, indicating that SN inappropriate assigns valence to emotionally salient stimuli (Lee et al., 2014). Additionally, certain genes that impart increased risk of developing schizophrenia have been associated with decreased striatal volume and hyperconnectivity within frontostriatal loops (Shepherd, 2013).

Of note, insular-SN connectivity can discriminate patients from healthy controls (Mikolas et al., 2016; Wang X. et al., 2016). One recent publication associated the severity and development of psychosis with the extent of hypoconnectivity throughout SN CSTC circuitry, specifically between the left AI, the bilateral putamen, and caudate nucleus (Wang C. et al., 2016). Further, Wang C. et al. (2016) demonstrated that individuals with schizophrenia displayed reduced structural and functional integrity within CSTC tracts of the SN. Reductions in network integrity may also explain interrupted information processing observed in individuals with schizophrenia, which is experienced as abnormal sensory processing (White et al., 2010).

### Eating Disorders (EDs)

EDs affect about 0.5% of women and can cause significant physical and psychological burden; for example, anorexia nervosa (AN) has the highest mortality of any psychiatric disorder (Hudson et al., 2007). EDs are characterized by altered self-image and maladaptive eating behaviors, and include AN, bulimia nervosa (BN) and binge-eating disorder (BED; McClelland et al., 2013). In recent years, functional imaging studies of EDs have pinpointed the intersection of cognitive and reward systems as integral to eating behavior regulation (Val-Laillet et al., 2015).

One systematic review of 10 structural MRI studies suggested that compared to healthy controls, both decreased and increased gray matter volumes in a variety of frontal brain areas characterize AN and BN, respectively, and may normalize with successful treatment (Van den Eynde et al., 2012). AN and BN patients show reduced gray matter volume specifically in SN regions including the caudate nucleus, anterior cingulate cortex and insula, among other areas (Schäfer et al., 2010; Friederich et al., 2012; Frank et al., 2013; Coutinho et al., 2015). On DTI, AN patients show abnormal thalamic connectivity to the dlPFC and anterior PFC (Frieling et al., 2012; Hayes et al., 2015), and reduced fractional anisotrophy in the medial dorsal thalamic radiations (Biezonski et al., 2015; Hayes et al., 2015). Further, increased structural connectivity between the insula and striatum is observed in ED (Frank et al., 2016; Shott et al., 2016).

On task-based fMRI, BN patients display low frontostriatal activity on a number of cognitive control tasks, including the Simon Spatial task (Marsh et al., 2009b, 2011) and Go/No-Go (Skunde et al., 2016). Similarly, AN patients have also been shown to have altered frontostriatal activation on executive function tasks, including the Wisconsin Card Sorting Task (Lao-Kaim et al., 2015), and delay discounting (Wierenga et al., 2014; Decker et al., 2015). During reward conditioning, AN patients show abnormally high SN activity relative to controls (Frank et al., 2012), and an increased preference for delayed rewards over immediate rewards. Dorsal caudate dysfunction is also associated with AN patients in a number of tasks, including a monetary choice task (Bischoff-Grethe et al., 2013; Bailer et al., 2016), and food-cue processing (Sanders et al., 2015). This increase in striatal reactivity may be related to a number of trait-based or neurochemical factors, including abnormal trait anxiety (Bailer et al., 2016), harm avoidance (Bailer et al., 2013), obsessive thoughts (Rothemund et al., 2011) and striatal dopaminergic receptor availability (Bailer et al., 2013, 2016; Broft et al., 2015).

Both AN and BN patients have altered SN function during disease-relevant stimuli. In one study, presenting food-related visual cues to individuals with either BN or BED resulted in increased ACC and insula activation, and BN patients reported higher levels of arousal (Schienle et al., 2009). In another study, BN participants displayed low activity in the insula to food images, while AN showed higher activity in the caudate and insula (Brooks et al., 2011). Another study showed that AN participants show abnormally high activation to aversive taste in the insula and putamen (Cowdrey et al., 2011). Abnormal ACC-insular resting state activity has also been observed in ED patients (Amianto et al., 2013; Dunlop et al., 2015b). Altered connectivity strength and path length between the insula and thalamus has been observed in AN (Geisler et al., 2016), as well as decreased functional connectivity between the thalamus, putamen and insula (Ehrlich et al., 2015). Given all this evidence, the variation in symptoms across different classes of EDs—for example, the presence of binging behavior in BN and BED, but not AN—make it difficult to assess convergent implicated brain regions, and both structural gray matter and functional activity analyses in these populations should be expanded (Schäfer et al., 2010).

There is evidence that corticostriatal loop circuits are affected in EDs. Specifically, individuals with EDs often display impaired self-regulatory control—this includes inhibition of both motor and emotional responses—that can be traced to dysfunction within dorsal frontostriatal circuits crucial for self-regulation (Berner and Marsh, 2014). Authors who recently reviewed the balance between incentive reward and inhibitory circuits in EDs (Wierenga et al., 2014) hypothesized that different patterns of disordered eating represent a spectrum of regulatory capability, ranging from extreme cognitive control in AN to deficiencies of cognitive control in BN. The dorsal neural system supporting such regulatory capacity—which includes functions of inhibition, emotion regulation, and goal-directed behavior—includes the dorsal caudate, dACC and insula, among other SN regions (Wierenga et al., 2014).

### Summary

Both structural and functional abnormalities within and between corticostriatal loop circuits are associated with psychiatric pathology. Generally, these disorders can be characterized by an inability to exert cognitive control over maladaptive thoughts, impulsive behaviors or attention to appropriate salient internal and external stimuli; for example, OCD involves disrupted control mechanisms, but lacks the element of disturbed motivational salience that is well defined in MDD and SUD. Regardless of clinical phenotype, consistent implication of the dACC, AI and dorsal striatal nodes in psychiatric etiology suggests that the SN-CSTC loop is indeed crucial for psychological health and adaptive functioning across a variety of disorders.

## Targeting the SN-CSTC with Therapeutic Brain Stimulation

**Figure 3:**
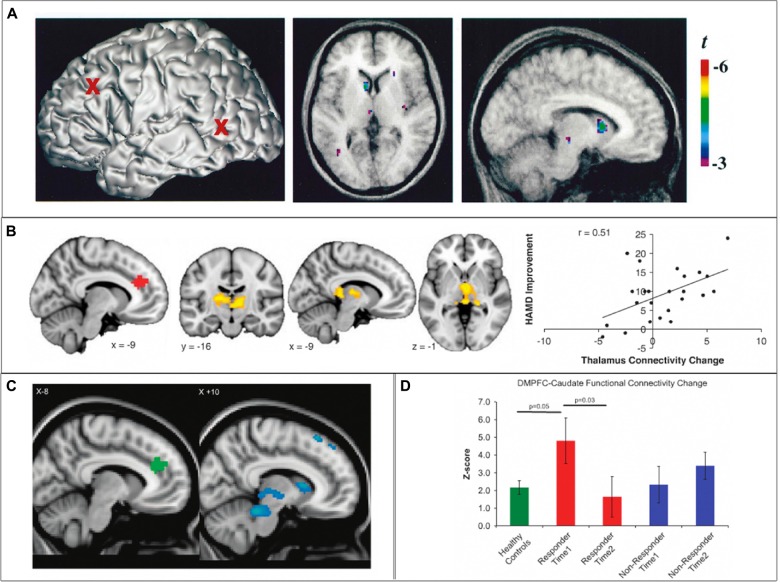
**SN cortico-striatal-thalamo-cortical circuit engagement during therapeutic brain stimulation. (A)** In healthy controls, 10 Hz repetitive transcranial magnetic stimulation (rTMS) of the left dorsolateral prefrontal cortex (dlPFC) results in increased dopamine transmission in the ipsilateral caudate nucleus and thalamus. **(B)** In depressed individuals, clinical improvement following 10 Hz dmPFC-rTMS was correlated with increased connectivity between the dACC and the thalamus. **(C,D)** In obsessive-compulsive disorder (OCD) patients, clinical improvement following 10 Hz dmPFC-rTMS was correlated with decreased functional connectivity between the dACC and the caudate nucleus, thalamus, putamen and midbrain. Adapted from **(A)** Strafella et al. (2001); **(B)** Salomons et al. (2014); **(C,D)** Dunlop et al. (2016).

For individuals with psychiatric disorders, the mainstays of conventional treatment are psychotherapy and pharmacotherapy. However, these approaches are ineffective for a substantial proportion of patients. For example, an estimated one third of MDD patients do not respond to 2–4 sequential trials of pharmacotherapy or psychotherapy (Rush et al., 2006). Therapeutic brain stimulation is an emerging alternative in cases where conventional approaches fail (Figure 3). A number of techniques for therapeutic brain stimulation are entering clinical use for treatment-resistant psychiatric illnesses. These include deep brain stimulation (DBS; Lozano and Lipsman, 2013), repetitive transcranial magnetic stimulation (rTMS; Lefaucheur et al., 2014) and transcranial direct current stimulation (tDCS; Tortella et al., 2015).

For the neuroscientist, these techniques can serve a dual role as both an intervention and a probe for investigating human brain function, either in health or in psychiatric illness. It is possible to assess the neurophysiological effects of brain stimulation using neuroimaging techniques, complemented by electrophysiological recordings. As described above, many clinical features of psychiatric illnesses are rooted in altered activity within relevant brain networks. Given that brain stimulation treatments are usually targeted to a specific brain region, the goal of such a treatment is to normalize regional cortical and downstream network activity.

The SN has been proposed as a key target for neuromodulation treatments across a variety of psychiatric illnesses (Downar et al., 2016; Dunlop et al., 2016). Stimulation-driven changes in the SN-CSTC loop could restore capacity for cognitive control via changes in network activity, resulting in symptom reduction (Veit et al., 2012; Sale et al., 2015). With respect to SN nodes and their downstream regulatory loops, both invasive and non-invasive brain stimulation techniques appear capable of modulating the activity of these circuits by imposing long-term changes in cortical nodes that cascade through the SN loop. These changes are accompanied by alterations in affect and behavior in the patient receiving treatment (Di Filippo et al., 2009).

In this section, we review the effects of DBS, rTMS and tDCS on cortico-striatal-thalamo-cortical circuitry through the SN specifically, such as they are known at the present time. We also review the available evidence to date about how changes in SN-CSTC function relate to the therapeutic effects of neuromodulation therapies in psychiatric illness.

### Deep Brain Stimulation (DBS)

DBS involves neurosurgical implantation of stimulation electrodes in target regions of the brain under stereotaxic guidance. The implanted electrodes modulate abnormal neural activity by applying electric fields that stimulate adjacent axons, resulting in the modification of electrical communication within connected functional brain networks (Kühn and Volkmann, 2016; Lin et al., 2016; Rachid, 2016). Although this technique is invasive, expensive and requires subspecialist expertise, DBS does carry the advantage of being able to directly target the deeper nodes of CSTC circuits, such as the striatum, pallidum, subthalamic nucleus or brainstem dopaminergic structures, as well as the white matter tracts connecting these nodes.

Some of the earliest uses of DBS were in modulating the activity of motor CSTC loops to treat movement disorders, including essential tremor and Parkinson’s disease (PD; Chen et al., 2013); the latter remains the most common therapeutic application of DBS (Fasano and Lozano, 2015). However, more recently, DBS has begun to show promise as a treatment for a number of neuropsychiatric disorders (Williams and Okun, 2013), including MDD (Mayberg et al., 2005; Schlaepfer et al., 2014), OCD (Lipsman et al., 2013a) and AN (Lipsman et al., 2013b).

For DBS in psychiatric disorders, the stimulation targets are generally found in CSTC circuits outside of motor loop. In MDD, for example, targeting the subgenual cingulate cortex (sgCC) may have modulatory downstream effects on the dACC by propagating stimulation effects through the rostral cingulate cortex (Morishita et al., 2014). Further, sgCC-DBS has normalized hypoactivity of the dlPFC and ACC, among other areas, in depressed patients without any observed cognitive side effects (Mayberg et al., 2005). More generally, sgCC-DBS appears to be an effective intervention for severe, treatment-resistant MDD. Follow-up studies and meta-analyses suggest that overall, sgCC-DBS may continue to ameliorate depressive symptoms over time, with persistence of beneficial effect for over a decade in some cases (Giacobbe et al., 2009; Morishita et al., 2014). It also appears that response rates to ongoing DBS stimulation may improve over time, with benefits accruing and persisting over periods of several years among responders (Kennedy et al., 2011).

Thus far, DBS has not been used to target the SN-CSTC loop directly in MDD. However, DBS targeting the nucleus accumbens (NAcc) has been successful in some cases of treatment-resistant depression, with responders showing improvements in hedonic capacity (Bewernick et al., 2010). PET imaging in these cases demonstrated that stimulation of the NAcc yielded reductions in metabolic activity in the “reward CSTC loop” projecting to vmPFC and frontal pole. This finding illustrates the potential of DBS to modulate (i.e., suppress) activity in CSTC circuits for therapeutic effect.

One illustrative case study suggests that DBS targeting the SN-CSTC may not necessarily exert desirable effects in MDD (Stefurak et al., 2003). In this report, a patient with intractable PD (and a remote history of a major depressive episode) underwent implantation of DBS electrodes bilaterally in the subthalamic nucleus. Activation of one electrode yielded the expected improvement in the tremor of the contralateral upper limb. However, activation of the other electrode had the unexpected effect of inducing an intense dysphoria of rapid onset. The patient described feeling “similar in some respects to my depression but a thousand times worse… Someone could have come in to shoot me and I could not have cared less”. The effect was reliably reproducible, and mood returned rapidly to baseline with cessation of stimulation. When functional neuroimaging was performed, stimulation of one electrode yielded reductions in supplementary motor area activity, alongside improvement of the contralateral tremor. Stimulation of the other electrode yielded suppression of activity in the SN regions including the dACC as well as the caudate nucleus, accompanied by rapid descent into dysphoria. This case illustrates the feasibility of using DBS to modulate the SN-CSTC loop circuit, and also illustrates that such modulation can be accomplished via deep subcortical targets such as the subthalamic nucleus. However, at the same time, it illustrates that suppression of SN activity may impair rather than improve mood regulation, with deleterious rather than beneficial effects in the setting of MDD.

The suppressive effects of subcortical DBS on CSTC loop functions may be of more benefit in the setting of OCD. DBS targets in OCD may include the ventral striatum, sgCC, NAcc or the medial forebrain bundle (Lipsman et al., 2013a; Williams and Okun, 2013). Although these targets lie outside the main nodes of the SN-CSTC loop, there is some evidence that the therapeutic effects of DBS in OCD ensue from modulation of activity in SN sites. Specifically, a case series of NAcc-DBS in OCD reported considerable inter-individual variability in the degree of clinical improvement; resting-state fMRI revealed that the degree of symptom improvement correlated to the degree of reduction in functional connectivity between the stimulation target (NAcc) and two nodes of the SN: the dACC and the dlPFC (Figee et al., 2014). Indeed, it has been suggested that all of the major DBS targets in OCD exert therapeutic effects by modulating activity in the ACC and associated regions of the striatum (Bourne et al., 2012).

Subcortical DBS for psychiatric disorders is thought to influence the regulatory activity of CSTC loops. Investigations of DBS to central thalamic regions in neurological disorders have identified several effects of stimulation, including increased D2 dopamine receptor concentration in the striatum, improved functional connectivity between the thalamus and striatum, and modulated plasticity in the striatum (Bourne et al., 2012). These effects are hypothesized to contribute to improvements in striatal plasticity, resulting in increased regulatory capacity over cognition and learning (Lin et al., 2016).

### Repetitive Transcranial Magnetic Stimulation (rTMS)

rTMS is a non-invasive brain stimulation technique that alters neural excitability by delivering focused magnetic field pulses to cortical areas non-invasively through the skull (Hallett, 2007). Repeated trains of pulses can produce durable increases or decreases (depending on the stimulation pattern) in the strength of the synapses of the stimulated neurons, via the mechanisms of long-term potentiation and depression (Karabanov et al., 2015). The durable effects of rTMS were initially noted via facilitation of motor evoked potentials with repeated stimulation of the primary motor cortex. Subsequently, it was found that rTMS delivered to the dlPFC improved mood in patients with depression (George et al., 1995; Pascual-Leone et al., 1996). Over the following 20 years, dozens of studies and several meta-analyses have established high-frequency left, low-frequency right and bilateral dlPFC-rTMS as superior to sham stimulation in the treatment of major depression (Berlim et al., 2013b,c; Berlim and Van Den Eynde, 2014; Gaynes et al., 2014). rTMS is now approved and used clinically as a treatment for medication-resistant depression in a variety of jurisdictions around the world (Lefaucheur et al., 2014; Milev et al., 2016; Perera et al., 2016).

rTMS is also showing promise as a treatment for a variety of other psychiatric disorders characterized by hypofunctioning of the SN. Supportive meta-analyses are now available for rTMS in treating SUD (Gorelick et al., 2014; Dunlop et al., 2016), PTSD (Berlim and Van Den Eynde, 2014), bipolar disorder (McGirr et al., 2016) and OCD (Berlim et al., 2013a; Trevizol et al., 2016).

The stimulation targets used in these studies typically correspond to frontal nodes of the SN. For example, the dlPFC region showing greatest efficacy in MDD has been reported to be a region anticorrelated to the sgCC, which also corresponds well to the SN’s dlPFC node (Fox et al., 2012). Stimulation of the dACC and adjacent dmPFC has also been employed in MDD (Bakker et al., 2015; Kreuzer et al., 2015), and stimulation of medial SN nodes in the dACC/dmPFC and adjacent pre-supplementary motor area have also shown promising effects in OCD (Mantovani et al., 2010; Dunlop et al., 2015a) and PTSD (Isserles et al., 2013). In healthy controls, there is evidence that rTMS of SN nodes such as the dlPFC or dmPFC/dACC can enhance or inhibit impulse control as measured on a delay-discounting task (Cho et al., 2010, 2015; Figner et al., 2010), and improve cognitive processing on executive functioning tasks (Esslinger et al., 2014), suggesting a generalized effect on cognitive control. Taking together these lines of evidence, recent reviews have suggested that the therapeutic effects of rTMS may be best understood not as “antidepressant” *per se*, but more generally as enhancement of cognitive control via improved SN integrity (Downar et al., 2016; Dunlop et al., 2016). This would account for its transdiagnostic efficacy across a range of psychiatric disorders involving cognitive control deficiency (McTeague et al., 2016).

The pertinent issue for the purposes of this review article is whether the therapeutic mechanisms of rTMS involve modulation of SN-CSTC loop circuits. Two questions thus arise: first, does rTMS of cortical targets cause neurophysiological changes in the CSTC loop for that target; and second, do these changes (if present) correlate to the behavioral and clinical effects of rTMS?

Regarding the first question, the neurophysiological mechanisms of rTMS are complex and still under investigation; accounts have been proposed at various levels of explanation from genetic and cell-molecular processes, to neurotransmitters and their receptors to synapses, to micro- and macro-level network connectivity changes (for reviews, see Noda et al., 2015). However, several convergent lines of evidence suggest that rTMS does indeed cause neurophysiological effects not merely at the stimulation site, but also throughout its CSTC loop circuit, targeting the entire loop in a precise, well-demarcated fashion. For example, PET studies using the D2 receptor tracer 11C-raclopride have shown that rTMS of the left primary motor cortex induces dopamine release specifically in the ipsilateral putamen, in a region corresponding to the known projection zone for corticostriatal projections from the primary motor cortex; no changes were seen in other striatal regions such as the caudate nucleus, NAcc or the contralateral putamen (Strafella et al., 2003). Another study by the same group, using the same tracer, demonstrated that rTMS of the left dlPFC induced dopamine release specifically in the head of the ipsilateral caudate nucleus, but not the NAcc, putamen or contralateral caudate nucleus (Strafella et al., 2001). A subsequent PET-rTMS study with a D2/D3 tracer demonstrated that rTMS of the dmPFC/dACC induced dopamine release in a circumscribed region of the dorsal putamen and underlying globus pallidus (Cho et al., 2015).

fMRI-rTMS studies support the premise that rTMS pulses activate not only the target region of cortex but also its associated striatal partner. fMRI-rTMS studies in healthy controls have directly demonstrated that rTMS pulses to the frontopolar cortex caused BOLD activations in both the frontal pole and in the ventral striatum; rTMS pulses to the dlPFC, in contrast, caused BOLD activations in both the dlPFC and the dorsal caudate nucleus (Hanlon et al., 2013, 2016). A session of inhibitory, continuous theta-burst stimulation to the frontopolar cortex reduced the BOLD response to individual pulses at the same target, with the effect seen prominently in ventral striatum (Hanlon et al., 2015). Thus, the available neuroimaging evidence from fMRI and PET studies suggests that rTMS directly activates not only the stimulation site but also the associated subcortical loop circuit, in a circumscribed fashion; these activations are accompanied by changes in dopamine neurotransmission in the subcortical projection zones of the stimulation target. rTMS of SN cortical targets (i.e., the dlPFC) appears to modulate activity in the subcortical components of the SN-CSTC loop circuit as well.

Regarding the second question of whether these changes in SN-CSTC loop circuit function are related to the behavioral and clinical effects of rTMS, several lines of evidence are now supportive. In a PET-rTMS study in healthy controls, an inhibitory form of rTMS (continuous theta-burst stimulation) delivered to the left dlPFC impaired performance on the Montreal Card Sorting Task, a set-shifting task requiring the cognitive control functions of the SN. This impairment was associated with reduced dopamine release in a circumscribed region of the head of the caudate nucleus (Ko et al., 2008). Likewise, in the previously mentioned PET study of dmPFC-rTMS (Cho et al., 2015), rTMS-induced changes in dopamine release in the globus pallidus showed a U-shaped relationship to change in impulsivity, as indexed on the delayed discounting task. Thus, the effects of rTMS on SN-CSTC loop circuits appear to translate into effects on cognitive control capacity.

There is also growing evidence that the therapeutic effects of rTMS in psychiatric illness may be mediated by changes in SN-CSTC loop circuit integrity. One supportive study used resting-state fMRI to examine CSTC functional connectivity in MDD patients who underwent a course of rTMS directed at the dmPFC/dACC. In this study, patients with low baseline functional connectivity from the dACC to the putamen (a region corresponding to that identified in the PET study of dmPFC-rTMS by Cho et al., 2015) and MD thalamus showed a greater degree of clinical improvement, and the degree of clinical improvement correlated to increases in functional connectivity from the stimulation target (dACC) and the MD thalamus (Salomons et al., 2014). The finding of dACC CSTC functional connectivity as both a predictor and correlate of clinical improvement was replicated in a follow-up study in ED patients undergoing dmPFC-rTMS for binge and purge behaviors (Dunlop et al., 2015a). In another follow-up study in patients with OCD (Dunlop et al., 2016), connectivity from the dACC to the head of the caudate nucleus and the MD thalamus was also a predictor and a correlate of clinical improvement; however, *reductions* rather than increases in connectivity within this CSTC loop were required for clinical improvement, paralleling the findings of Figee et al. (2014) in OCD patients undergoing DBS (as discussed in the previous section). These findings suggest that the therapeutic effects of rTMS in MDD, ED and OCD may be mediated by changes in the integrity of the SN-CSTC circuit. Future studies will be required to determine whether these findings also apply to the more commonly employed protocol of dlPFC- rather than dmPFC-rTMS.

### Transcranial Direct Current Stimulation (tDCS)

tDCS is another non-invasive brain stimulation technique that uses scalp electrodes to deliver mild (1–2 mA) electrical currents to target brain regions, thereby modulating ongoing brain activity (Blumberger et al., 2015). The mechanisms of tDCS and related techniques such as transcranial alternating current stimulation (tACS) are still under investigation and debate (for reviews, see Nitsche et al., 2008; Tortella et al., 2015). However, from a clinical perspective, the technique is attractive for offering a favorable profile of safety, tolerability and low cost. For this reason, tDCS is under active investigation not only as a research tool but also as a potential therapeutic intervention in psychiatric illness (Tortella et al., 2015). The best-studied indication to date has been MDD, with several randomized controlled trials published; recent meta-analyses of these trials have found tDCS to be more effective than sham stimulation, with an effect size comparable to antidepressant medications (Meron et al., 2015; Brunoni et al., 2016).

Lateral cortical nodes of the SN are widely used as tDCS targets, both in basic science and clinical studies. One of the most common targets for tDCS in the literature to date has been the dlPFC, often operationalized as EEG sites F3 and F4 in the standard 10–20 montage. The F3 site has been shown to correspond fairly closely to the dlPFC node appearing in the SN (Mir-Moghtadaei et al., 2015), suggesting that tDCS of F3 and F4 is anatomically positioned to stimulate the SN. Research studies and clinical trials of tDCS have targeted the dlPFC due to its hypothesized central role in executive function and cognitive control (Kuo and Nitsche, 2012). tDCS targeting this region has been shown to improve performance across the domains of working memory (Fregni et al., 2005), impulsivity (Fecteau et al., 2007) and social cognition (Knoch et al., 2008). tACS has also been performed with electrodes placed bilaterally over the dlPFC (EEG sites F3 and F4) and its parietal counterpart sites (EEG sites P3 and P4), with one study reporting frequency-dependent enhancements of lucid dreaming during REM sleep (Voss et al., 2014).

The dlPFC has also been a tDCS target in the clinical treatment of substance use and ED. It is hypothesized that stimulating the dlPFC enhances top-down control over maladaptive eating behaviors and substance consumption, and suppresses cravings generated by dysfunctional reward circuitry (Fregni et al., 2005; Lapenta et al., 2014). Indeed, tDCS of the dlPFC has been shown to reduce cravings, consumption and behavioral impulsivity in long-term smokers (Rachid, 2016), which may represent effects in attentional salience or inhibition networks (Fregni et al., 2005; Lapenta et al., 2014).

An important question for the purposes of this review article is whether tDCS actually stimulates any of the deeper nodes of CSTC circuits, or whether its effects are confined to superficial cortical regions. A purely cortical mechanism of localized changes in synaptic plasticity is often postulated in the literature, given that the relatively weak electrical fields employed in tDCS may not penetrate beyond the cortex (Nitsche et al., 2008). However, several recent findings suggest that tDCS may indeed modulate neural activity in *subcortical* structures of the CSTC loop circuits. First, a study using resting-state fMRI found that tDCS of the primary motor cortex increased its functional connectivity to the thalamus, and additionally increased connectivity between the parietal cortex and the caudate nucleus (Polanía et al., 2012). Second, a study using the MRI-based perfusion technique of arterial spin labeling (ASL) demonstrated that anodal right and cathodal left dlPFC-tDCS caused decreases in resting perfusion of the head of the caudate nucleus as well as the medial and lateral orbitofrontal cortex (Weber et al., 2014). Finally, a recent study using magnetic resonance spectroscopy (MRS) has for the first time directly demonstrated that active but not sham dlPFC-tDCS (anodal left, cathodal right) increases levels of glutamate and glutamine in the striatum as well as N-acetylaspartate in the dlPFC itself (Hone-Blanchet et al., 2015). Taken together, these findings suggest that tDCS can indeed modulate both neurotransmission and network connectivity patterns in the subcortical as well as the cortical components of CSTC loops when targeting the SN (i.e., via the dlPFC).

So far, little information is available that speaks to whether the therapeutic mechanisms of tDCS in psychiatric illness ensue via modulation of CSTC loop circuits, as appears to be the case for DBS and potentially for rTMS. As of this writing, the literature on the effects of tDCS on CSTC activity in general remains very limited (as reviewed in the preceding paragraph). Alternative possibilities are that the therapeutic effects of tDCS ensue purely through modulation of cortico-cortical network connectivity, or conceivably through local modulation of synaptic connections under the cortical stimulation site alone. Future studies of therapeutic tDCS in psychiatric illness will need to incorporate not only larger sample sizes, but also more detailed measures of cognitive control capacity, as well as neuroimaging observations before and after treatment, in order to identify the behavioral and neural correlates of clinical improvement. Such studies may help to determine whether tDCS of SN nodes enhances cognitive control, and if so, whether this enhancement ensues via modulation of the SN-CSTC loop circuits.

## Unresolved Questions and Future Directions for Study

The emerging picture from the evidence reviewed in the previous three sections is that: (i) the SN-CSTC loop may play a critical role in the voluntary engagement of cognitive control; (ii) abnormalities of cognitive control are a common, pervasive and transdiagnostic feature of many psychiatric illnesses; (iii) these transdiagnostic deficits of cognitive control may arise from abnormalities of functioning within a specific member of the brain’s many CSTC loops—namely, the CSTC loop serving the SN; and (iv) emerging brain stimulation therapies such as DBS, rTMS and tDCS exert neurophysiological effects on targeted CSTC loop circuits, and these effects may be central to the mechanisms by which they alleviate psychiatric illness.

At the moment, this account of the CSTC loop through the SN must be considered preliminary, with many findings requiring further replication and study, and many questions still outstanding. In this final section, we review some potentially fruitful directions for further study.

### Particular Contributions of the SN-CSTC Loop to Voluntary Cognitive Control

At present, it would be helpful to have a clearer understanding of the SN’s role in cognitive control. Specifically, more information should be generated about the contributions of the SN to self-regulation of cognition, emotion and behavior in healthy brain function. The particular contributions of the SN should be distinguished from contributions of other functional networks involved in executive function, such as the neighboring frontoparietal networks known as dorsal attention or central executive networks. It would also be helpful to have a clearer understanding of the distinct roles of the SN vs. its immediately posterior counterpart comprising the posterior insula and mid-cingulate cortex. Finally, it is necessary to better understand how engagement of the subcortical projection sites of the SN (in the head of the caudate, globus pallidus, MD thalamus, and rostral substantia nigra) relates to the voluntary vs. passive engagement of cognitive control functions.

### Abnormalities of SN-CSTC Loop Integrity and Function in Psychiatric Illness

In parallel with the previous theme, it would be helpful to have more detailed descriptions of the abnormalities present in the functional integrity, structural integrity and neurochemistry of the SN-CSTC loop circuit in the major categories of psychiatric illness. fMRI, ASL, DTI, VBM and PET studies will all be useful in this regard. The “intervention-probe” properties of DBS, rTMS, and tDCS may also yield useful information of a causal rather than correlational nature. It will be helpful to understand which psychiatric disorders share SN-CSTC dysregulation as a common feature, and whether there are disorders (for example, AN) where such pathology is relatively absent. Finally, as relatively few studies to date have explored the neural heterogeneity of illness among individuals with MDD, PTSD, OCD, SUD or other Axis I disorders (as defined by the DSM-IV), it will be helpful to understand whether SN-CSTC pathology is a pervasive feature across most individuals *within* each disease category, or whether instead only a certain sub-population of patients within MDD, PTSD, OCD or SUD show abnormalities of SN-CSTC functioning. The latter case, if true, may help to explain the problematic heterogeneity of outcomes currently seen with brain stimulation therapies, and may lead to methods for predicting which individuals are the best candidates for treatments targeting the SN-CSTC loop circuits. Better characterization of individual patients’ pathology using the Research Domain Criteria (e.g., cognitive control, response selection and response inhibition) may be useful in this regard (Morris and Cuthbert, 2012).

### Contributions of Other CSTC Circuits to Psychiatric Illness

Although mounting evidence suggests that SN-CSTC pathology may be a common feature of many psychiatric disorders, it is also clear that such pathology is far from the *only*, or even the most important, pathological feature in many individuals. For example, pathology of the “reward” or “incentive” CSTC loop from the NAcc to the vmPFC and frontal pole increasingly appears to be important across a variety of disorders. Aside from the well-established example of SUD, pathology of this loop may be important in OCD, MDD (particularly for the symptom of anhedonia), schizophrenia, and other psychiatric disorders (as noted throughout the previous three sections of this review). In addition, interactions (such as mutual inhibition) between the activity of the dorsal striatal-SN loop and the ventral striatal reward loop may be important for understanding psychiatric pathology and its heterogeneity; such interactions between dorsal and ventral CSTC loops are an increasingly prevalent theme of study in recent work combining neuroimaging and neurostimulation in psychiatric illness (for example, Liston et al., 2014; Hanlon et al., 2016).

### Effects of Brain Stimulation Techniques on SN-CSTC Function

The available literature to date provides mounting evidence that brain stimulation treatments are capable of modulating the activity of CSTC circuits, and that these effects may be central to the therapeutic properties of such treatments. Nonetheless, further evidence is needed to demonstrate and characterize the effects of brain stimulation treatments on CSTC function—to some extent for DBS, more so for rTMS, and especially so for tDCS and tACS. As stated above, a variety of techniques will be useful in this regard: in addition to fMRI studies of network integrity, PET and MRS—and potentially voltammetry—will be useful for characterizing neurochemical effects, while electrophysiological recordings (noninvasive MEG and EEG, and invasive intracortical recordings when available) will be essential in assessing the direct effects of stimulation on neural activity. Much of the available literature to date has focused on motor CSTC loops rather than other prefrontal loops. However, in light of the increasing popularity of brain stimulation in treating psychiatric illness, more attention is due to the SN-CSTC loop in future study.

### Optimizing Brain Stimulation Protocols for Modulating SN-CSTC Function

The optimal parameters for therapeutic DBS, rTMS and tDCS/tACS in psychiatric illness are still being refined, and in many cases are entirely unknown. To date, very few studies have sought to optimize the frequency, intensity, protocol, inter-session interval or even dose (i.e., session number) for therapeutic brain stimulation as a primary aim. The assessment of relative efficacy in most cases has been empirical, based on clinical measures (e.g., standardized symptom scales) that may not be well suited to capturing the nuanced effects of therapeutic brain stimulation. For example, after 20 years of clinical trials of rTMS in MDD, there is a wealth of evidence about crude response and remission rates for this heterogeneous population, but very little evidence about *which* types of patients respond best to treatment, or whether responders are characterized specifically by deficiencies of cognitive control rather than conventional mood symptoms *per se*, as proposed in this review. A more nuanced outcome measure (behavioral, cognitive or neurophysiological) may be helpful in providing a benchmark for optimizing the parameters of stimulation. For example, if the mechanism of effect for dlPFC- or dmPFC-rTMS does indeed depend upon enhancements in deficient cognitive control and SN-CSTC loop circuit integrity, then obtaining markers of cognitive control and SN integrity will be essential to any parameter-optimization study. Candidate markers might include behavioral measures such as performance on flanker or delayed discounting tasks, electrophysiological measures such as coupling between theta and gamma oscillations, or neuroimaging markers such as D2 receptor occupancy on PET or SN-striatal-thalamic functional connectivity on fMRI. Future studies of therapeutic brain stimulation may need to make use of such markers in order to make progress in exploring the many dimensions of stimulation parameters that are still awaiting optimization. Future studies involving fMRI should also consider the false-positive rate of fMRI in the application of past research, and study design and processing (Eklund et al., 2016); this point highlights the importance of replication studies and meta-analyses in developing new rTMS targets and in interpreting the effects of rTMS on brain function.

## Conclusion

Converging evidence suggests that, of the brain’s many CSTC loop circuits, the specific circuit serving the SN may be of particular relevance to cognitive control, and of transdiagnostic relevance to psychiatric pathology. This proposal, if supported by future work, is of more than purely academic interest. With the emergence of anatomically selective brain stimulation technologies as therapeutic tools, it is becoming possible to target specific CSTC loop circuits of the brain in an increasingly precise manner, and to modulate their activity in a variety of ways. Therapeutic brain stimulation of the SN-CSTC loop circuit may constitute a method for directly targeting the underlying pathophysiology of several types of psychiatric illness, or at least a subpopulation of individuals within these categories of illness. Stimulation targeting other CSTC loops, such as those through the ventral striatum, may further expand the range of disorders and individuals whose illness is amenable to brain stimulation treatments. With a better understanding of CSTC function in health and psychiatric disease, it may become possible to tailor the target and parameters of stimulation to the individual, depending on the underlying pathology. This individualized, brain-based approach to psychiatric treatment would constitute an important step forward in addressing the daunting prevalence and burden of mental illness around the world.

## Author Contributions

SKP, KD and JD wrote and edited the manuscript and figures.

## Funding

SKP has received funding from the University of Toronto Ontario Graduate Fund. KD has received funding from the Canadian Institute for Health Research (CIHR) Vanier Scholarship. JD has received research support from CIHR, NIH, the Klarman Family Foundation, the Buchan Family Foundation and the Toronto General and Western Hospital Foundation. He has also received a travel stipend from Lundbeck and from ANT Neuro, and in-kind equipment support for an investigator-initiated study from Tonika/Magventure.

## Conflict of Interest Statement

The authors declare that the research was conducted in the absence of any commercial or financial relationships that could be construed as a potential conflict of interest.
